# Epidemiology of hip fracture in Iraq and development of a country-specific FRAX model

**DOI:** 10.1007/s11657-025-01631-3

**Published:** 2025-11-26

**Authors:** Ahmed Abdulbari, Nizar A. Jassim, Yasir H. Motlaq, Hassan A. Ismael, Waleed Arif Tawfeeq Al Ani, Chra Kawa M. Nafai, Ali Abdulrahman Younis, Taha Ahmed Qaradaghi, Farah Jaafar Mahdi, Abbas Mahdi Rahmah, Alaa Hussein Alameri, Fareed Alsudany, Zaid W. Al-Shahwani, Tariq Jassim Mohammed, Mohannad Khalil Ahmed AbuKhumrah, Adra Abdul Ruda Kadum, Asal Adnan, Marwan Z. Yahya, Nicholas C. Harvey, Mattias Lorentzon, Eugene McCloskey, John A. Kanis, Helena Johansson

**Affiliations:** 1 Rheumatology & Rehabilitation Department, Ibn Sina Training Hospital, Baghdad, Iraq; 2 Iraqi Board for Medical Specializations, Baghdad, Iraq; 3 Radiology Department, Ibn Sina Training Hospital, Baghdad, Iraq; 4 Public Health Department, Ibn Sina Training Hospital, Baghdad, Iraq; 5 Department of Medicine, College of Medicine, Mustansiriyah University, Baghdad, Iraq; 6 Rizgary Teaching Hospital, Erbil, Iraq; 7 College of Medicine, University of Mosul, Ibn Sina Teaching Hospital, Mosul, Iraq; 8 Sulaimanyiah Internal Medicine Teaching Hospital, Sulaimanyiah, Iraq; 9 College of Medicine, Mustansiriyah University, Baghdad, Iraq; 10 Mustansiriyah University, National Diabetes Center, Baghdad, Iraq; 11 Health Statistics and Geographic Information System Department, Ministry of Health, Baghdad, Iraq; 12 Orthopedic Department, Ibn Sina Training Hospital, Baghdad, Iraq; 13 Iraqi Board for Orthopedic Specialty, Baghdad, Iraq; 14 Alkarkh General Hospital, Baghdad, Iraq; 15 Ibn Sina Training Hospital, Baghdad, Iraq; 16 Department of Internal Medicine, College of Medicine, Rheumatology Unit, University of Baghdad, Baghdad, Iraq; 17 Emergency Department, Ministry of Health, Baghdad, Iraq; 18 MRC Lifecourse Epidemiology Centre, University of Southampton, Southampton, UK; 19 Sahlgrenska Osteoporosis Centre, Institute of Medicine, University of Gothenburg, Gothenburg, Sweden; 20 Division of Clinical Medicine, School of Medicine & Population Health, University of Sheffield, Sheffield, UK; 21 Centre for Metabolic Diseases, University of Sheffield Medical School, Sheffield, England

**Keywords:** FRAX, Fracture probability, Epidemiology, Hip fracture, Iraq

## Abstract

***Summary*:**

Hip fracture data were retrieved from the Iraqi government’s Ministry of Health and the Kurdistan region’s Ministry of Health for the years 2022–2023 and used to create a FRAX® model to facilitate fracture risk assessment. Hip fracture rates and probabilities were compared with estimates from neighboring countries.

**Introduction:**

There are no published data on the epidemiology of osteoporotic fractures in Iraq. This paper describes the epidemiology of femoral fractures in Iraq and the development of the corresponding country-specific FRAX® tool for the prediction of fractures.

**Methods:**

Femoral fracture data (ICD-10 S72) were retrieved from the Iraqi government’s Ministry of Health and the Kurdistan region’s Ministry of Health for the years 2022–2023 in Iraq. The age- and sex-specific incidence of hip fracture in Iraqi residents and national mortality rates were used to create a FRAX® model. Fracture probabilities were compared with those from neighboring countries having existing FRAX models.

**Results:**

Fracture rates were low and comparable to those in neighboring countries, with hip fracture rates closest to estimates from Saudi Arabia at older ages. Ten-year fracture probabilities were lower in males than in females and were comparable to those in neighboring countries.

**Conclusion:**

The FRAX model should enhance the accuracy of determining fracture probability among the Iraqi population and help guide treatment decisions.

## Introduction

The development of tools to assess fracture risk has enabled a step change in the management of osteoporosis, as patients can now be selected for treatment based on absolute fracture risk rather than bone mineral density (BMD) T-score alone. Of the several assessment tools available, the most widely used is FRAX®, which is recommended in more than 100 national and international guidelines [1]. FRAX calculates 10-year fracture probabilities in males and females from readily obtained clinical risk factors with or without bone mineral density (BMD) measurements at the femoral neck (https://www.fraxplus.org/). The algorithms in FRAX are based on a series of meta-analyses using primary data from population-based cohorts that examined a list of candidate clinical risk factors for fracture [2, 3]. The output of FRAX comprises the probability of a major osteoporotic fracture (hip, spine, distal forearm, or proximal humerus) or hip fracture alone. This probability is, in turn, dependent upon the risk of fracture and the competing risk of death, both of which vary from country to country [1]. Before FRAX, clinicians primarily relied on bone mineral density (BMD) measurements alone, which, while helpful, are not universally available [4, 5] and cannot capture the full spectrum of risk associated with osteoporotic fractures globally [6].

The wide range of fracture probability worldwide [7] demands that data for age-specific incidence of fracture and death should be available for the construction of country-specific FRAX models. Still, information on fracture incidence is frequently poor or absent. Notwithstanding, the availability of FRAX has stimulated studies of fracture incidence that can be used for the generation of new FRAX models; specific examples include Armenia, Belarus, Brazil, Kazakhstan, Mexico, Moldova, Russia, Turkiye, and Uzbekistan [8]. To date, a single study has addressed risk factors associated with hip fracture in Iraq, but there remains a dearth of information on hip fracture rates in Iraq [9]. The present study describes the epidemiology of hip fractures in Iraq and the development of a corresponding FRAX model.

## Methods

Iraq, a middle-income country in Southwest Asia, covers a total area of approximately 438,317 km^2^ with a 2024 population of 46.1 million [10]. The country comprises 19 governorates, including the autonomous Kurdistan Region in the north. Iraq's demographic profile includes a nearly equal gender distribution and a majority Arab population, with significant Kurdish and other ethnic minorities. Iraq’s healthcare infrastructure and data collection vary across regions, which is relevant for national fracture incidence estimation. It is bordered by Saudi Arabia to the south, Turkiye to the north, Iran to the east, the Persian Gulf and Kuwait to the southeast, Jordan to the southwest, and Syria to the west.

### Fracture incidence

Femoral fracture data ICD-10 code: S72; includes hip and shaft) were retrieved from the Iraqi government’s Ministry of Health and the Kurdistan region’s Ministry of Health for the years 2022-2023 in Iraq. The data comprised Iraqi nationals, and the data from the two regions were merged and stratified in 5-year age intervals in males and in females from the age of 40 years. The catchment population for each 5-year age interval for each year was supplied by the Iraqi Ministry of Health and the Kurdistan region’s Ministry of Health. The incidence of femoral fractures was computed from the number of fractures and the catchment populations in each 5-year age interval in males and in females. Fracture rates for Iraq were compared with those available in neighboring countries (Kuwait, Turkiye, Saudi Arabia, Iran, and Jordan) [11–14]. For other major osteoporotic fractures (MOF; clinical spine, forearm, and humeral fractures), it was assumed that the age- and sex-specific incidence ratios of these fractures to hip fracture risk found in Sweden were comparable to those in Iraq. This assumption has been used for many of the FRAX models with incomplete epidemiological information. Available information suggests that the age- and sex-stratified pattern of fracture is very similar in the Western world, Australia, and Eastern Europe [15–18].

### Fracture probability

The development and validation of FRAX have been extensively described [1, 2]. The risk factors used were based on a systematic set of meta-analyses of population-based cohorts worldwide and validated in independent cohorts with over 1 million patient-years of follow-up. The construct of the FRAX model for Iraq retained the beta coefficients of the risk factors in the original FRAX model, together with the incidence rates of femoral fracture and mortality rates for Iraqi nationals. National mortality rates for Iraqi nationals for the years 2022-2023 were obtained from the United Nations (UN) [19]. Ten-year fracture probabilities were compared to those of the neighboring countries where a FRAX model was available (Jordan, Kuwait, Saudi Arabia, Iran, and Turkiye).

To compare Iraqi hip fracture probabilities with those in other regions of the world, the remaining lifetime probability of hip fracture from the age of 50 years was calculated for males and females, as described by Kanis et al. [20]. In the present analysis, values for Iraq were compared with those for Abu Dhabi, Botswana, Bulgaria, Canada, China (Hong Kong), Denmark, Finland, France, Germany, Greece, Hungary, Iran, Kazakhstan, Kuwait, Moldova, Morocco, Netherlands, Poland, Portugal, Romania, Russia, Saudi Arabia, Singapore, South Africa, Spain, Sweden, Tunisia, Turkiye, UK, Ukraine, USA and Uzbekistan [21].

## Results

A total of 18,239 fracture cases were identified over the two-year interval. Femoral fracture rates were higher in females than in males for ages between 60 and 79 years. In females, the incidence was relatively constant from the age of 70 years and above table 1.

**Table 1:** Number of femoral fractures in 2022 and 2023, population at risk and annual incidence/100,000 with 95% confidence intervals (95%CI) in Iraq

Age	Number of hipfractures		Population 2022 and 2023		Incidence/100,000 and 95% CI			
	Men	Women	Men	Women	Men		Women	
40–44	1682	739	2,274,460	2,304,836	74	70–78	32	30–34
45–49	1382	738	1,680,909	1,730,793	82	78–87	43	40–46
50–54	1026	918	991,092	1,234,197	104	97–110	74	70–79
55–59	1073	882	1,126,712	1,179,067	95	90–101	75	70–80
60–64	950	1071	775,942	848,077	122	115–130	126	119–134
65–69	882	983	510,771	532,103	173	161–184	185	173–197
70–74	911	1255	333,235	335,326	273	256–292	374	354–396
75–79	617	860	175,535	211,499	351	324–380	407	380–435
>80	974	1296	217,292	315,834	448	421–477	410	388–433
Total	9497	8742	8,085,948	8,691,732	117	115–120	101	98–103

**Fig. 1:**
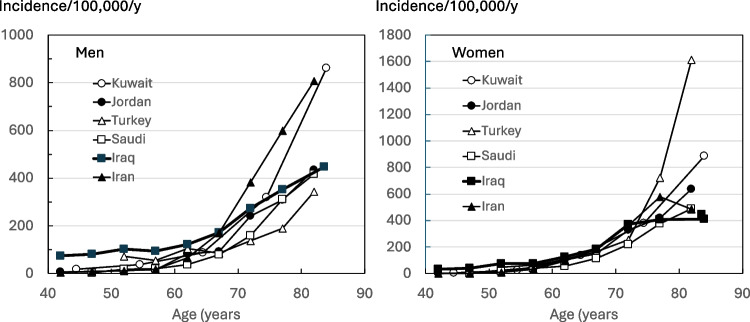
Ten-year probabilities of hip fracture (HF) and major osteoporotic fracture by age in Iraqi males and females

The comparison of annual fracture rates with neighboring countries is shown in Figure 1. Fracture rates were within the range reported from neighboring countries from the age of 65 years upwards and similar to the rates reported in Saudi Arabia for older ages. Below this age, rates were higher than in neighboring countries. It is relevant to note, however, that the Iraqi data refer to femoral fracture rather than hip fracture.

Lifetime probabilities for hip fracture at the age of 50 years are shown in Table 2. For Iraq, probabilities were approximately 1 in 18 for females, similar to probabilities in other Middle Eastern countries, and substantially lower than the majority of European countries.

**Table 2:** 10-year probabilities of hip fracture (HF)and major osteoporotic fracture (MOF) in men and women at age 65 years with a prior fracture and no other risk factors with unknown BMD in Iraq and neighbouring countries. Body mass index is set to 25kg/m^2^.

Country	Women			Men	
	MOF	HF		MOF	HF
Saudi Arabia	8.2	2.0		5.1	1.3
Kuwait	8.9	2.6		5.6	1.7
Jordan	9.8	2.7		5.8	1.6
Türkiye	10	2.3		5.8	1.4
Iran	11	3.6		6.4	2.1
Iraq	11	2.8		6.2	1.6

Comparative data for probabilities of hip fracture and major osteoporotic fracture for neighboring countries are illustrated for males and females with a prior fragility fracture (Table 2). There was reasonable consistency between countries at this age.

Table 2 10-year probabilities of hip fracture (HF)and major osteoporotic fracture (MOF) in males and females at age 65 years with a prior fracture and no other risk factors with unknown BMD in Iraq and neighboring countries. Body mass index is set to 25kg/m^2^.

**Fig. 2:**
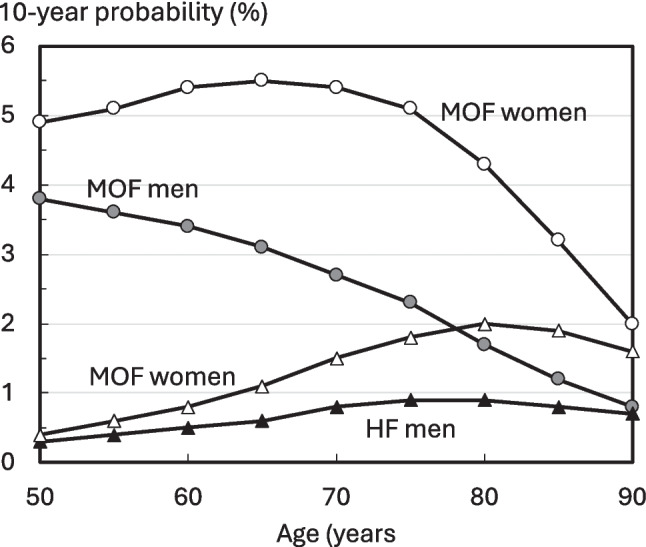
Annual incidence of femoral fracture (Iraq) and hip fracture in males (left-hand panel) and females (right panel) by age from Iraq, Jordan, Kuwait, Saudi Arabia, and Turkiye

10-year probabilities of a hip fracture are shown for males and females in Figure 2. Probabilities in the Iraqi female population rose with age up to the age of 80 years and plateaued thereafter due to the competing effect of mortality. A similar pattern of hip fracture probabilities was observed in men. The 10-year probabilities of an MOF declined in females above the age of 65 years, whereas in men, 10-year probabilities declined progressively with age Table 3.

**Table 3:** Life-time probability of hip fracture in the Iraqi population at the age of 50 years compared with selected countries. Data from [22] unless otherwise indicated

Country	Life-time risk at 50 years (%)	
	Women	Men
Sweden	25.6	11.0
South Africa (white)	23.4	7.7
Denmark	23.0	11.3
France	19.3	5.9
China (Hong Kong)	17.7	7.6
USA (Caucasian)	16.1	7.5
Türkiye	15.9	3.6
Canada	15.5	5.8
Greece	15.4	6.8
Uzbekistan	14.7	8.7
UK	14.4	5.0
Germany	14.2	5.3
Portugal	13.7	4.8
Finland	12.9	6.0
Jordan	12.8	6.1
Kazakhstan	12.6	6.0
Spain	12.6	4.2
Netherlands	12.5	5.4
Singapore (Indian)	12.5	5.2
Bulgaria	11.2	4.4
Qatar**	11.0	8.8
Hungary	10.8	4.2
Poland	10.1	4.2
Moldova	9.3	5.7
**Kuwait**	**9.2**	**7.6**
Abu Dhabi	8.9	8.1
Iran	8.3	5.5
Russia	7.7	3.8
Romania	7.0	3.8
USA (black)	5.9	2.7
Ukraine	5.6	2.9
Iraq*	5.5	4.2
Saudi Arabia**	4.6	3.7
South Africa (Black)	4.5	1.9
Morocco	4.1	3.1
Botswana	1.1	1.4
Tunisia	0.7	0.7

## Discussion

This study documented the incidence of femoral fractures in Iraq to inform the construction of a country-specific FRAX model. Fracture rates were higher in females than in males from the age of 60 years, but greater in males than in females below this age. From an international perspective, hip fracture incidence was low in both males and females [7]. It is of interest that the incidence of hip fracture was somewhat similar to that reported for Saudi Arabia and Kuwait. There were, however, differences in fracture probability between countries with advancing age. The explanation for the difference likely lies in the impact of mortality since fracture probability integrates the fracture hazard with the competing effect of mortality. These observations emphasize the importance of the death hazard as well as the fracture hazard in the determination of fracture probability.

There are several important considerations concerning the primary data. For Iraq, femoral fractures were identified (Classification of Diseases [ICD]−10 code S72 rather than hip fracture (ICD-10 codes S72.0, S72.1, S72.2). Thus, the incidence of hip fracture is overestimated, particularly at younger ages. From the age of 70 years, more than 90% of femoral fractures are hip fractures in males and females from Sweden, but at the age of 50 years, the proportion is approximately 75% [16]. This may explain in part the higher male-to-female incidence ratio under the age of 60 years and the relatively high rates at younger ages (see Figure 1). Secondly, it is notable that, although the fracture incidence rose with age, this plateaued in females from the age of 70 years. This raises the question of whether all older females with hip fractures come to hospital attention, as reported in several countries in Eastern Europe [22–25]. These considerations provide a research agenda, the results of which may update the FRAX model.

A minority of countries that have an FRAX model also have robust information on the risk of other major osteoporotic fractures. In the absence of such information, FRAX models assume that the age- and sex-specific pattern of these fractures is similar to that observed in Malmo, Sweden [16]. The assumption has been validated in studies from Canada [18], Iceland [17], the US [26], the UK [27], Australia [28], and Eurasia [15] despite very marked differences in the incidence of hip fracture [7]. This commonality of pattern is supported by register studies, which indicate that in those regions where hip fracture rates are high, so too is the risk of forearm fracture and spine fractures (requiring hospital admission) [29, 30].

The strength of the study lies in the 2-year study interval and data based on the national rather than a regional population. Nevertheless, the accuracy of the FRAX model is dependent on the accuracy of the fracture and death hazards used in the construction of the FRAX model. It is relevant, however, that accuracy errors have little impact on the rank order with which the FRAX tool categorizes risk in each population [23, 31]. Still, they do change the absolute number generated and thus have implications where treatment guidelines are based on cost-effectiveness or the economic burden of disease. A further limitation of the present study is that femoral fractures were identified rather than just hip fractures, so that rates are relatively higher at younger ages. This limitation will affect not only the probabilities of hip fracture, but also the probabilities of major osteoporotic fractures since the latter are derived from the former. Future studies require more granular characterization of fracture sites to remedy this deficit so that the FRAX model can be refined.

In summary, a FRAX model has been created for Iraq based on a national estimate of the incidence of hip fractures. The model should enhance the accuracy of determining fracture probability among the Iraqi population and help guide treatment decisions.
